# Selected Physicochemical and Pharmaceutical Properties of Poly-*ε*-caprolactone and Poly(d,l-lactide-*co*-*ε*-caprolactone) Conjugates of Lamivudine Synthesized via Ring-Opening Polymerization

**DOI:** 10.3390/polym11122124

**Published:** 2019-12-17

**Authors:** Tomasz Urbaniak, Witold Musiał

**Affiliations:** Department of Physical Chemistry and Biophysics, Pharmaceutical Faculty, Wroclaw Medical University, Borowska 211, 50-556 Wroclaw, Poland; tomasz.urbaniak@umed.wroc.pl

**Keywords:** drug–polymer conjugates, ring-opening polymerization, polymer physicochemistry, submicron drug delivery systems, drug release modification

## Abstract

The modification of drug fate after administration may be achieved by the covalent coupling of active pharmaceutical ingredients with macromolecules. To prolong or delay the release, slowly degrading polymers such as polyesters may be applied for conjugation. The detachment of a covalently conjugated drug from the polymeric matrix relies mostly on the material degradation profile and barely on the weak interaction between the drug and macromolecules. In the present study, lamivudine was conjugated via ring-opening polymerization with poly-*ε*-caprolactone and poly(d,l-lactide-*co*-*ε*-caprolactone). The influence of the reaction parameters on the course of the polymerization and physicochemical properties of obtained conjugates were investigated. Subsequently, selected reaction products were formulated into submicron particles, and drug release profiles in physiological-like conditions were investigated. The course of the reaction was monitored via gel permeation chromatography. The structure and physicochemical properties of products were evaluated via spectroscopic, calorimetric, and diffractometric methods. The profile of the drug release from particles prepared by the slow evaporation of conjugate solution from o/w emulsion was monitored with high-performance liquid chromatography. Both an elevated reaction temperature and higher catalyst concentration increased the polymerization rate and simultaneously promoted the side reactions, resulting in a broad molecular weight distribution of products in the range from 1.30 to 2.15. The physicochemical properties of conjugates obtained in different conditions varied and had a direct influence on the drug release. The release curve of lamivudine from particles based on low molecular weight conjugates achieved a plateau between 18.9 and 22.2 μg per mg of conjugate within a month. Drug detachment from particles composed of high molecular weight conjugates exhibited a distinct delay period preceded by a drug burst release at a maximal level of 13.3 μg per mg of conjugate. Conjugate chemical composition and the degree of crystallinity were also found to influence the release.

## 1. Introduction

A combination of the active pharmaceutical ingredient (API) and macromolecular species is a widely employed technique of drug modification. Poly(ethylene glycol) is presumably the most widely researched example of non-targeting linear polymers employed for drug conjugation [1]. Nevertheless, differently structured polymers, i.e., polyesters, polystyrene, or polyoxazolines may be employed as conjugating agents [2]. The binding of API to biomacromolecules exhibiting direct affinity to receptors present on aimed cells is another widely investigated strategy of the targeted drug delivery [3]. Along with the emerging advantages and possibilities provided by polymer-modified APIs, numerous requirements were set for these new therapeutic systems. Especially in the case of intravenously administered conjugates, features such as the biocompatibility, biodegradability, and non-toxicity are essential for safe therapies [4]. Furthermore, potential impurities present in materials after the polymerization process may trigger a negative cellular response. Therefore, the in vivo circulation time of a drug delivery system (DDS) should be controlled, e.g., via tuning of the carrier decomposition rate. In terms of carrier elimination, the molecular weight may be considered as one of the crucial factors. To extend the conjugate circulation time in the organism, high molecular weight molecules may be applied to prevent glomerular filtration. However, due to the risk of carrier accumulation and following adverse effects, the capability of controlled degradation and elimination are essential features during DDS design [5].

A large group of commercially applied, non-toxic biodegradable polymers are polyesters such as poly(*ε*-caprolactone) (PCL), polylactic acid (PLA), and their copolymers. In physiological conditions, PCL and PLA degrade via hydrolysis or the enzyme-catalyzed hydrolysis pathway [6]. Depending on the polymer molecular weight, complete material degradation may take from weeks up to years. Organic acids slowly released as the result of the degradation are introduced into the metabolic chains or eliminated unmodified [7]. The hydrolysis rate of polyester-based solid DDS depends primarily on the surface area to volume ratio, polymer molecular weight, and polymer chain arrangement [8]. Water necessary for the hydrolysis process penetrates the material more efficiently through amorphous regions; thus, the crystallinity degree is a relevant factor in terms of carrier degradation and drug release kinetics. Numerous researches employing PCL and PLA as materials for the fabrication of implants, tissue scaffolds [9,10], and parenterally administered particulate DDS [11,12] confirmed their long-term safety. Compatibility with pharmaceutical excipients and excellent biocompatibility make these materials promising substrates for DDS.

Both polymers are obtained mainly in the course of ring-opening polymerization (ROP) of cyclic monomers *ε*-caprolactone (*ε*-CL) and lactide (LA). In accordance with the reported mechanism of ROP catalyzed by metal–organic compounds, molecules bearing hydroxyl groups are capable of polymerization initiation, and are built into the polymer chain in the first steps of the reaction [13]. The employment of drugs as ROP initiators resulted in drug–polyester conjugates that are suitable for further processing into submicron drug carriers [14]. As it was described above, drug release from these materials can be extremely slow and may be modified by the degree of crystallinity, the chemical composition of polymer chains, and their molecular weight. All of these features may be controlled during drug-initiated polymerization. One of the most frequently applied catalysts in ROP is tin alkoxides, e.g., those accepted by the FDA as food additive tin (II) 2-ethylhexanoate (SO) [15]. The suggested mechanism of reaction includes the nucleophilic attack of molecules with hydroxyl groups on the monomer–SO complex. 

The present study aims to elucidate the influence of selected synthesis parameters on the physicochemical properties of drug–polymer conjugates. Four selected antimicrobial APIs employed in various macrophage-related diseases were applied as model drugs for conjugate synthesis. Due to the potentially long in vivo circulation time of solid conjugate-based micro-matrices, uptake by phagocytic cells is a most probable route of conjugate elimination. This phenomenon may be exploited as a macrophage-targeted drug delivery strategy for intracellular viral and microbial infections such as HIV, leishmaniasis, or tuberculosis [16,17,18]. Among evaluated reactions, lamivudine (LV)-initiated polymerization resulted in the formation of conjugates in a satisfying manner. Recent findings underline the role of macrophages as HIV reservoirs and their contribution in antiretroviral drug resistance [16]. Therefore, evaluated conjugates may serve as material for the preparation of antiretroviral LV delivery systems aiming at infected macrophages. The previously reported catalytic system for the synthesis of lamivudine-poly-*ε*-caprolactone via ROP [19] was employed in an extended variant to evaluate the influence of reaction temperature and catalyst concentration on the ROP kinetics and physicochemical properties of drug–polymer conjugates. Subsequently, LV release from selected drug-initiated ROP products formulated into submicron particles was investigated.

## 2. Results and Discussion

### 2.1. Lamivudine–Polymer Conjugate Synthesis

Products of ROP catalyzed by SO in the presence of selected reaction initiating APIs: clarithromycin (CLAR), rifampicin (RIF), acyclovir (AC), and LV were evaluated via spectroscopic methods. Subsequently, the most effective reaction initiated by LV was further investigated in terms of the reaction parameter influence on the kinetics of ROP as well as the product physicochemical properties.

#### 2.1.1. Structural Analysis

According to the suggested mechanism of ROP catalyzed by metal–organic compounds, molecules containing hydroxyl moieties may serve as reaction-initiating agents. In the stage of polymerization initiation, these molecules become covalently bound to the propagating polymer chains [20]. In the presented study, four antimicrobial, hydroxyl-bearing APIs administered in macrophage-related infections were applied as *ε*-CL ROP initiators. Products of *ε*-CL ROP in the presence of CLAR, RIF, AC, and LV, as well as products of *ε*-CL and LA copolymerization in the presence of LV, were evaluated via electrospray ionization time-of-flight mass spectrometry. Isotopic distributions of the peaks corresponding to the polymeric structures found by the experiment were compared to the patterns simulated for selected possible structures: drug–polymer conjugates, cyclic polymers, and unconjugated polymers with various positively charged adducts. Spectra analysis confirmed the presence of isotopic distributions separated by *m/z* values of 114 Da, which is equal to the molecular weight of one PCL subunit in all the performed reaction variants. Isotopic distributions separated by *m/z* values of 72 Da were observed exclusively in the copolymerization products. Even though the formation of polymeric structures occurred, the conjugate formation could not be confirmed in RIF, CLAR, and ACY-initiated reactions. Moreover, no other expected structures, namely unconjugated polymer chains or cyclic unconjugated polymers, could be identified. The formation of unknown polymeric structures in reactions performed in the presence of RIF, CLAR, and ACY suggest drug degradation in applied conditions and subsequent reaction initiation by drug decomposition products. The observed instability of the mentioned drugs in the reaction environment made them inappropriate for further investigation. A promising candidate, LV, was subsequently evaluated in *ε*-CL homopolymerization and the random copolymerization of *ε*-CL and LA in varying reaction conditions. In the case of LV-initiated reactions, particular isotopic distributions of high intensity could be matched to isotopic distributions simulated for LV–polymer conjugates or the other expected polymeric structures. A summary of the obtained isotopic distributions matched to corresponding simulations of isotopic distributions for polymeric structures obtained in reactions performed in the presence of LV is presented in Table 1.

The presence of isotopic distributions of *m/z* values corresponding to *m/z* values of LV–polymer conjugates confirms covalent bonding between drug and polyester chains. In several spectra, observed isotopic distributions could be matched to unconjugated free PCL or PLA chains, which suggests the occurrence of polymerization initiated by a trace amount of water present in the reaction system.

#### 2.1.2. Influence of Synthesis Parameters on Polymerization Reaction Course

**Figure 1:**
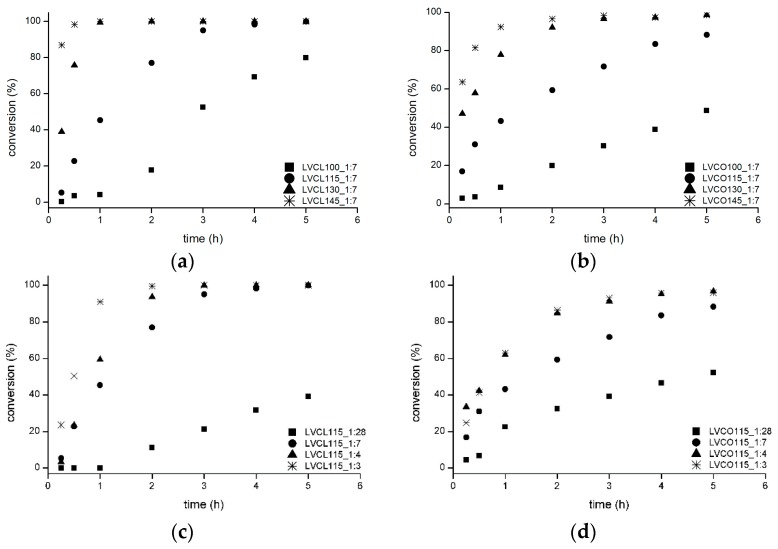
Monomer conversion versus reaction time for (**a**) *ε*-caprolactone (*ε*-CL) homopolymerization and (**b**) LA-*ε*-CL copolymerization in varying temperatures ranging from 100 °C to 145 °C; (**c**) *ε*-CL homopolymerization and (**d**) LA-*ε*-CL copolymerization in varying 2-ethylhexanoate (SO) concentrations expressed as SO:LV ratios; a detailed explanation of the synthesis abbreviations is included in the Table 1 header.

The ROP initiated by LV molecules was further evaluated to assess the influence of reaction temperature and catalyst concentration on the polymerization process. Monomer conversion on the course of ROP conducted in varying temperatures (Figure 1a,b) and catalyst concentrations (Figure 1c,d) were investigated concurrently with a chromatographic evaluation of molecular weight values.

During 5 h of reaction mixture heating after the injection of SO, complete monomer conversion was achieved in a few reaction variants. The considerably faster rate of monomer consumption was observed in reactions employing lower SO:LV ratios of 1:4 and 1:3, which resulted in a reduction of time required to deplete the available monomers. Increased polymerization rates in reactions employing higher SO concentrations are in line with the suggested reaction mechanism and were observed in studies evaluating analogous reaction systems [21,22]. Temperature elevation from 100 °C to 145 °C also resulted in faster depletion of the monomer. Such an effect was described in ROPs employing various metal–organic catalysts and monomers [23,24]. This is presumably due to the increased molecule mobility and reduced viscosity of the melted reaction mixture. The absence of monomers in the reaction mixture during the period after complete monomer conversion, both in homopolymerization and copolymerization, suggests that the highest employed temperatures were below the reactions’ ceiling temperatures.

**Figure 2:**
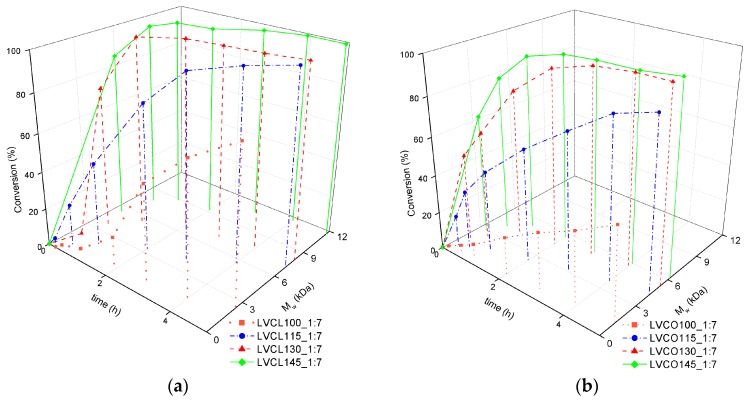
Monomer conversion vs. time vs. weight average molecular weight for (**a**) *ε*-CL homopolymerization and (**b**) LA-*ε*-CL copolymerization reactions employing an SO:LV ratio of 1:7, conducted in temperatures of 100 °C, 115 °C, 130 °C, and 145 °C; a detailed explanation of the synthesis abbreviations is included in the Table 1 header.

The weight average molecular weight M_w_ of ROP products was monitored concurrently with monomer conversion. The influence of reaction temperature on the M_w_ changes with respect to reaction time and monomer conversion is depicted in Figure 2.

**Figure 3:**
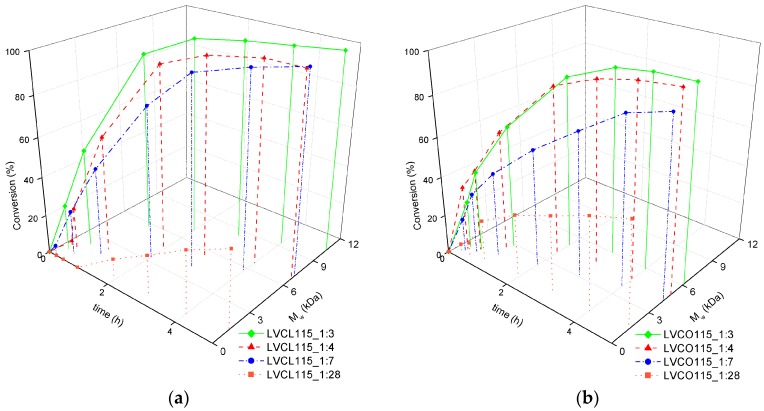
Monomer conversion vs. time vs. weight average molecular weight for (**a**) *ε*-CL homopolymerization and (**b**) LA-*ε*-CL copolymerization reactions conducted in 115 °C and varying SO concentrations expressed as the SO:LV ratio; a detailed explanation of the synthesis abbreviations is included in the Table 1 header.

During the course of *ε*-CL homopolymerization conducted in higher temperatures of 130 °C and 145 °C, M_w_ tends to increase even after the depletion of available monomers. In other reaction variants, the molecular weight remains constant, which suggests the completion of polymerization and a lack of further side reactions. Transesterification side reactions were reported in ROPs performed in higher temperatures, especially in reactions employing SO as a catalyst [22]. Most of the described side reactions resulted in chains scission and an overall reduction of product molecular weight [25]. Nevertheless, M_w_ increasing side reactions such as the chain intermolecular condensation of unconjugated polymer chains with conjugates may occur in elevated temperatures and prolonged reaction times [26]. The analogous effect was observed in homopolymerization conducted in 115 °C in the presence of the lowest SO:LV ratio (Figure 3a).

The increased amount of highly hygroscopic catalysts in reactions employing lower SO:LV ratios provided an additional amount of water molecules that are capable of reaction initiation and follow the formation of pure PCL. Such unconjugated chains could be introduced into conjugate chains in further stages of the reaction [26]; thus, an M_w_ growth effect could be observed even in lower temperatures. A well pronounced induction period was observed in a homopolymerization reaction performed in the presence of the highest SO:LV ratio. The molecular weight of the *ε*-CL and LA copolymerization products did not alter after the complete monomer conversion in reactions LVCO115_1:3 and LVCO115_1:4, which indicates a lack of side reactions observed in *ε*-CL homopolymerization (Figure 3b). No induction period was observed in all the performed copolymerization reactions, which suggest more efficient LA conversion at the initial stages of ROP.

**Figure 4:**
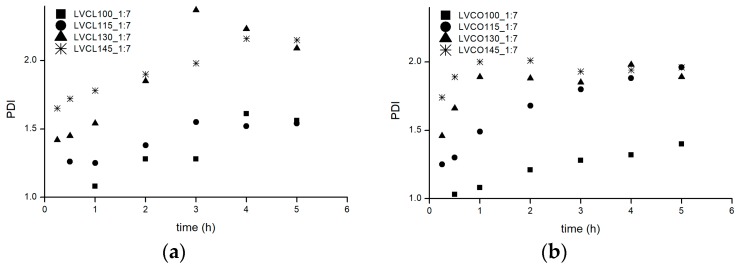

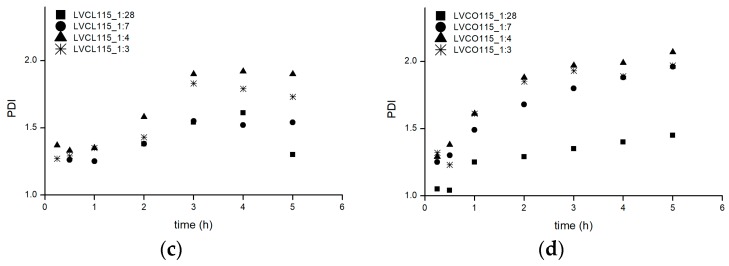
Molecular weight polydispersity index versus reaction time for: (**a**) *ε*-CL homopolymerization and (**b**) LA-*ε*-CL copolymerization in varying temperatures ranging from 100 °C to 145 °C; (**c**) *ε*-CL homopolymerization and (**d**) LA-*ε*-CL copolymerization in varying SO concentrations expressed as SO:LV ratio; a detailed explanation of the synthesis abbreviations is included in the Table 1 header.

Changes in the molecular weight polydispersity index (PDI) values over the course of all the evaluated reactions were monitored as well (Figure 4). In accordance with the above-mentioned results suggesting M_w_ growth occurring after monomer conversion, analogous PDI growth was observed in homopolymerization reactions conducted at higher temperatures of 130 °C and 145 °C and in the reaction system employing the lowest SO:LV ratio. The broadening of chain length distribution may potentially result in intermolecular transesterification and condensation reactions [20].

Final PDI values of ROP products obtained after complete monomer conversion were high, which suggests poor reaction control. The homopolymerization conducted with an SO:LV:*ε*-CL reactant ratio of 1:7:438 in 115 °C resulted in an acceptable PDI value of 1.54 and an M_w_ value of 7.08 kDa; this value is close to the expected molecular weight of 7.31, which was calculated from the employed monomer/initiator molar ratio. In the applied reaction setup, the lower reaction temperature and intermediate catalyst amount allowed a reaction performance in a reasonable time with sufficient control.

**Figure 5:**
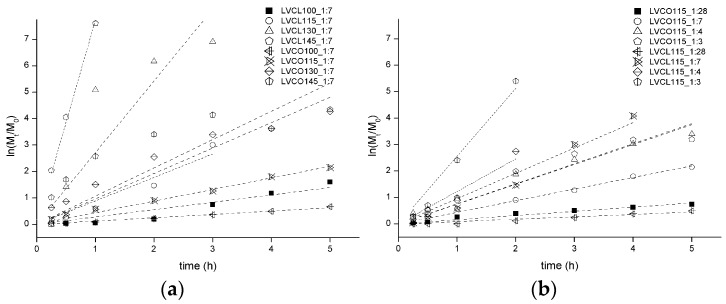
Ln(M_t_/M_0_) versus reaction time plot for *ε*-CL-LA copolymerization and *ε*-CL homopolymerization reactions conducted in (**a**) various temperatures and (**b**) SO:LV ratios; a detailed explanation of the synthesis abbreviations is included in the Table 1 header.

The kinetics of homopolymerization and copolymerization reactions in varying temperatures and SO:LV ratios were investigated. Plots of ln ([M_t_]/[M_0_]) versus reaction time t were fitted to linear functions (Figure 5a,b). At the presented graphs, [M_t_] is the percent of initial monomer concentration [M_0_] at a given reaction time t. The linear dependence observed on plots confirms first-order polymerization propagation with respect to monomers in nearly all the performed reactions except for LVCL115_1:4, LVCO145_1:7, LVCL 100_1:7, and LVCL130_1:7. Obtained data suggest the living character of polymerization in reaction periods before monomer depletion.

The R-square values of data linear fitting in four reaction variants were below 0.95 (Table 2). The initial flat fragment of the LVCL 100_1:7 kinetic curve indicates the occurrence of the polymerization induction period, which was reported in some *ε*-CL ROP variants [27]. The non-linearity of LVCO145_1:7 and LVCL130_1:7 kinetic curves suggests a decrease in the number of propagating polymer chains and is presumably the result of termination side reactions, occurring at later reaction stages. The increase of the reaction rate constants with growing temperatures and a decreased SO:LV ratio confirm that both evaluated variables influence the rate of LV-initiated polymerization of LA and *ε*-CL.

**Figure 6:**
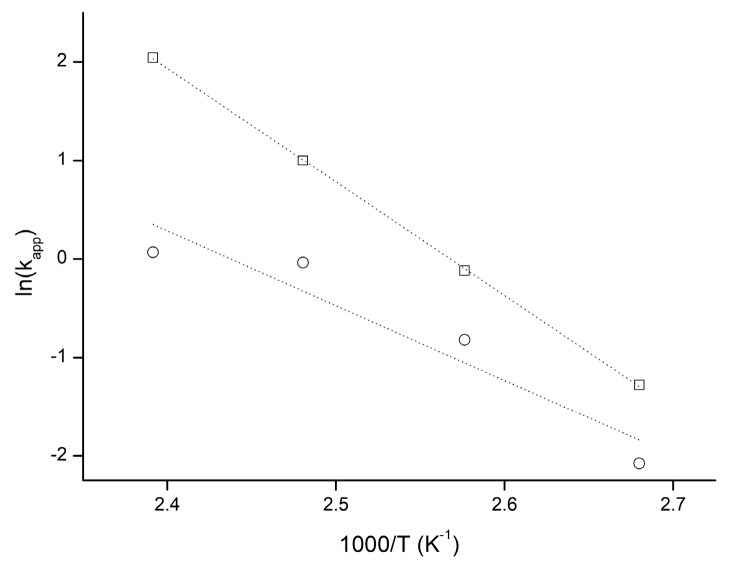
Arrhenius plot for LV-initiated homopolymerization (○) and copolymerization (□) reactions.

The apparent reaction rate constants calculated for the range of temperatures were used in the Arrhenius plot to calculate overall polymerization activation energy E_a_ (Figure 6). The E_a_ values calculated for the homopolymerization and copolymerization reactions were 95.86 ± 0.79 kJ mol^−1^ and 63.08 ± 14.27 kJ mol^−1^ respectively, which are close to the values reported for SO-catalyzed ROPs [22,28].

#### 2.1.3. Physicochemical Properties of Obtained Conjugates

Selected properties of products of LV-initiated ROP, terminated after 5 h, were investigated (Table 3). PCL-based conjugates were semi-crystalline with melting points oscillating around 60 °C. The observed melting temperatures and degrees of crystallinity are in good agreement with values reported for PCL synthesized in various procedures [29,30]. Observed variations in these properties are presumably the result of slightly different recrystallization conditions. Poly(d,l-lactide-*co*-*ε*-caprolactone) conjugates were amorphous and did not exhibit melting points. A random distribution of atactic LA monomers prevented the formation of a crystalline fraction. Minor crystalline peaks were observed in two samples; it is likely that these were formed due to the presence of longer *ε*-CL sequences that formed crystalline phase regions during the recrystallization process. The presence of these organized domains in this particular sample should be attributed to small differences in the purification procedure. Most probably they are not the result of conjugate chemical structure variability. The ratio between LA and *ε*-CL in copolymerization variants in which the monomers were completely converted was close to the monomer ratio employed in reaction mixtures. A higher content of LA monomers was present in samples obtained in ROP variants terminated before complete monomer conversion, due to the low reaction temperature or low SO concentration. This suggests that LA monomers are consumed more efficiently at the initial period of ROP.

### 2.2. Drug Release

In order to investigate drug release from obtained materials, five selected LV conjugates were formulated into the submicron particles. Micromatrices were obtained via the solvent evaporation technique, which is a method commonly employed in pharmaceutical applications [31]. A good size homogeneity and the spherical shape of all particles obtained via this technique assures that drug release is affected mainly by the physicochemical properties of the employed material. The hydrodynamic diameters and size polydispersity indexes (PDI_Hd_) of particle batches measured via dynamic light scattering are summarized in Table 4.

**Figure 7:**
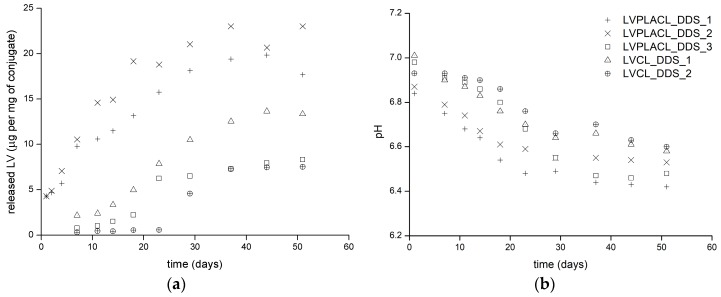
(**a**) LV release in phosphate-buffered saline at 37 ± 2 °C for period of 51 days and (**b**) corresponding changes of release media pH during release experiment.

Drug release from obtained particles in physiological-like conditions was evaluated for 51 days. The concentration of the released drug (Figure 7a) and pH increase of the release media resulting from polymer hydrolysis were monitored (Figure 7b). 

Formulations LVPLACL_DDS_1 and LVPLACL_DDS_2 based on low molecular weight poly(d,l-lactide-*co*-*ε*-caprolactone) conjugates released the drug in a nearly logarithmic manner with a plateau achieved approximately one month after the release experiment. Presumably, low molecular weight enabled fast chain degradation, while the amorphous structure of the polymers allowed effective water permeation through the polymer matrix [32]. These factors resulted in the fastest drug release rate among the evaluated formulations. The slightly faster release rate was observed in the preparation of LVPLACL_DDS_2 employing a conjugate with higher LA content. Pure hydrophobic PCL degrades significantly slower in comparison to pure PLA; therefore, the higher LA content in poly(d,l-lactide-*co*-*ε*-caprolactone) enabled faster hydrolytic chain scission. A pH decrease in the case of both formulations started from the first days of the experiment and achieved plateau at approximately the same moment, as drug release was completed. A slightly slower release and respective pH decrease were observed in formulation LVCL_DDS_1, employing a low molecular weight LV-PCL conjugate. This is most probably due to the semi-crystalline structure of the polymer matrix, which hampered water penetration, as well as due to the slower hydrolysis of hydrophobic PCL chains. Moreover, a slight delay in drug release and pH drop was recorded. Formulations LVPLACL_DDS_3 and LVCL_DDS_2 employing conjugates of higher molecular weight released LV with a distinctive delay period of approximately 14 days for the poly(d,l-lactide-*co*-*ε*-caprolactone) conjugate and 24 days for the homopolymer conjugate. The observed difference may be attributed to the different water penetration rates through semi-crystalline LV-PCL and amorphous poly(d,l-lactide-*co*-*ε*-caprolactone) conjugates. The delayed release may be the consequence of the polyester degradation profile resulting from water penetration to the polymer matrix and autocatalytic hydrolysis driven by the pH increase in the polymeric structure interior. This phenomenon is described as “auto-accelerated degradation”, and it could explain the rapid drug release and decrease in medium pH after an initial induction period [5]. The differences between the final maximum drug concentrations achieved in experiments evaluating different conjugates occurred due to different molecular weights and PDIs of conjugates. The same amount of conjugate was employed in each release experiment; therefore, the number of drug molecules was lower in preparations employing conjugates of higher molecular weight. Consequently, conjugates with four times higher molecular weight released approximately four times lower an amount of incorporated drug. Another factor that could influence drug release from obtained particles is the variability of their hydrodynamic diameters. The delayed release of LV from formulations LVPLACL_DDS_3 and LVCL_DDS_2 may be partly a result of the higher hydrodynamic diameters and consequently smaller particle surface per gram of particles exposed to the release medium. 

Dozens of particle formulations incorporating various drugs in PCL and PLA matrices were investigated in recent decades [33]. Submicron systems based on these polyesters tend to release API within hours/days, while the employment of larger micro-sized structures can prolong release up to weeks/months [34]. A considerable part of investigated systems suffers from the burst release effect, which is usually an unwanted phenomenon limiting their therapeutic suitability. LV-loaded PCL submicron particles were evaluated by Tshweu et al. [35]. Drug release from 10 kDa PCL matrices lasted approximately four days, with the initial burst release phase during the first hours of the experiments. An immensely different drug discharge profile from particles of comparable size based on the same polymer of similar molecular weight demonstrates the significant impact of covalent drug conjugation to the polymeric matrix. Another investigated LV-loaded particle-based DDS included poly(lactic-*co*-glycolic acid) submicron particles [36], polymethacrylic acid nanoparticles [37], and lipid nanoparticles [38]. All the reported systems released the drug within hours after the introduction to physiological-like conditions. Delayed drug release from the polymer structures covalently linked with API may be beneficial in terms of the aforementioned drug delivery via phagocytosis by macrophages. A lack of drug release after particle administration would provide the time necessary for particle transport to the site of action and subsequent uptake by the target cells. Ultimately, the administered drug dose would reach target cells resulting in increased therapy efficiency and the alleviation of side effects.

## 3. Materials and Methods 

### 3.1. Materials

The following materials were used in the study: *ε*-caprolactone (purity 97%, Sigma Aldrich, Darmstadt, Germany), calcium hydride (purity 95%, Sigma Aldrich), caprolactone (purity 97%, Sigma Aldrich), lamivudine (secondary pharmaceutical standard, purity 100%, Sigma Aldrich), clarithromycin (purity 100%, Sigma Aldrich), acyclovir (purity 100%, Sigma Aldrich), rifampicin (purity 100%, Sigma Aldrich), tin 2-ethylhexanoate (purity 92.5–100% Sigma Aldrich), poly(vinyl alcohol) (31 kDa, degree of hydrolysis 86.7–88.7%, Roth, Zielona Góra, Poland), dichloromethane (purity 98.5%, Chempur, Piekary Śląskie, Poland), methanol (purity 99.5%, Chempur), CDCl_3_ (purity 100%, Sigma Aldrich), tetrahydrofuran (purity 99.8%, Chempur), acetonitrile (purity 99.9%, Sigma Aldrich), ammonium acetate (purity 97–100%, Chempur), glacial acetic acid (purity 99.5%, Chempur), polystyrene standards (analytical standard grade, Sigma Aldrich), sodium azide (purity 99.0%, Sigma Aldrich), and phosphate saline buffer (0.01 M phosphate buffer, 0.0027 M potassium chloride and 0.137 M sodium chloride, pH 7.4, purity 99.9%, Sigma Aldrich).

### 3.2. Conjugate Synthesis

Bulk ring-opening drug-initiated *ε*-CL homopolymerization and *ε*-CL-LA copolymerization procedures were carried out in a three-necked flask equipped with a magnetic stirrer and reflux condenser in a dry nitrogen atmosphere for 5 h. E-caprolactone was dried over calcium hydride and distilled under reduced pressure, LA was recrystallized twice from ethyl acetate prior use. For the *ε*-CL homopolymerization reactions, 0.027 M of monomer was used, and for the *ε*-CL and LA copolymerization reactions, 0.013 M and 0.005 M respectively were used. In each synthesis, a mixture of monomer and initiator were preheated to the determined temperature before the injection of SO. LV, CLAR, RIF, and ACY were applied as potential reaction initiators. ROP kinetics were investigated in variants employing lamivudine as the initiator. The influence of reaction temperature and the amount of the SO on the reaction course was investigated. The ratio between monomers and LV in the homopolymerization and copolymerization reaction were constant in order to enable an assessment of the SO concentration impact. Samples of each reaction mixture were collected in seven intervals and evaluated via gel permeation chromatography. Crude products were dissolved in dichloromethane and recrystallized from cold methanol, dried, characterized, and stored under vacuum until further use. The molar ratio of reactants and polymerization temperatures were summarized in Table 5.

### 3.3. Gel Permeation Chromatography

The number average molecular weight and weight average molecular weight of samples were determined with gel permeation chromatography. Chromatograms were obtained with use of the Thermo Scientific high-performance liquid chromatography set, Dionex Ultimate 3000 (Thermo Scientific, Waltham, MA, USA) equipped with Phenogel 10^3^ A° column (Phenomenex, Torrance, CA, USA) in tetrahydrofuran in room temperature. The obtained M_w_ and M_n_ values relative to polystyrene standards were corrected according to the correcting coefficient of 0.56 [39]. Monomer conversion was estimated on the basis of the relative area of monomer and polymer-derived peaks.

### 3.4. Proton Nuclear Magnetic Resonance 

The proton nuclear magnetic resonance analysis of copolymerization products was performed on an ARX 300 MHz NMR spectrometer (Bruker, Billerica, MA, USA) in chloroform-d at 25 °C. Obtained spectra were used exclusively to estimate the ratio between monomers building poly(d,l-lactide-*co*-*ε*-caprolactone) chains. Peak integrals of LA proton-derived multiplet present at 5.10 ppm and *ε*-CL proton-derived triplet at 4.05 ppm were employed in calculations.

### 3.5. Electrospray Ionization Time-of-Flight Mass Spectrometry 

The formation of the drug–polymer conjugates in ROP was evaluated with electrospray time-of-flight mass spectrometry in acetonitrile on a micrOTOF-Q mass spectrometer (Bruker Daltonics, Bremen, Germany) in acetonitrile/chloroform mixture. Isotopic distributions of the peaks found by the experiment were compared to corresponding distributions of drug-tagged polymeric chains simulated by Mmass software (Free Software Foundation, Boston, MA, USA).

### 3.6. X-ray Powder Diffraction

Powder X-ray diffraction patterns were collected on a D2 Phaser (Bruker, Karlsruhe, Germany) diffractometer, operating at 30 kV and 10 mA, with a CuKa radiation and LYNXEYE detector. The samples were scanned over a 2θ range of 10–36° with a step size of 0.02° and step time of 0.5 s. The crystallinity degree was calculated with Diffrac Suite Eva software (Bruker AXS, Karlsruhe, Germany) from the ratio between the area of crystalline peaks and the total area of a diffractogram.

### 3.7. Differential Scanning Calorimetry

The melting temperatures of obtained conjugates were estimated from endothermic peaks in differential scanning calorimetry (DSC) curves. The DSC curves of conjugates were obtained using a DSC 214 Polyma (Netzsch, Selb, Germany) heat flux-type calorimeter. Measurement control and data analysis were performed with Proteus software (Netzsch, Selb, Germany). Samples for the DSC measurements were sealed in 40-ll standard aluminum crucibles with a single hole punched in the lid. The total mass of a sample was between 4 and 6 mg. An empty crucible of the same type was used as a reference. The DSC cell was purged with a stream of high-purity nitrogen (99.999%) at a rate of 25 cm^3^ min^−1^. DSC scans of all the samples were run at a heating rate of 5 °C min^−1^ in the temperature range of 0–150 °C.

### 3.8. Particle Preparation and Drug Release

In order to provide a comparable surface area to volume ratio of materials obtained in selected ROPs, conjugates were formulated into microspheres via the *o/w* emulsion solvent evaporation technique. Emulsion was obtained by the homogenization of 5 mL of 0.3 *w/v*% dichloromethane conjugate solution with 25 mL of distilled water 1 *w/v*% poly(vinyl alcohol) solution with the use of a laboratory rotor-stator homogenizer X120 (Ingenieurbüro CAT, Ballrechten-Dottingen, Germany) for 7 min with a 25,000 rpm homogenization rate. After emulsification, samples were left under magnetic stirring to 350 rpm for 3 h in room temperature in order to evaporate the volatile organic phase. Drug release from obtained particles after exposition to physiological-like conditions (phosphate buffer saline, pH 7.4; ionic strength of 162.7 mM; temperature of 37 °C) was evaluated. Particles were resuspended in release medium supplemented with 0.02% NaN_3_ in 37 °C and stirred gently for a period of 51 days. Samples of release medium were collected at least eight times during the whole experiment, filtered through 0.22-μm membrane filters, and evaluated via high-pressure liquid chromatography.

### 3.9. Dynamic Light Scattering

Particle hydrodynamic diameters and PDI_Hd_ values were evaluated via dynamic light scattering measurements on a Zetasizer Nano apparatus (Malvern, Worcestshire, UK) in phosphate-buffered saline. Each sample was measured four times, and the hydrodynamic diameters are expressed as mean ± SD.

### 3.10. pH Measurements

pH of the medium in drug release experiments was measured with an IJ44C pH electrode (Ionode, Folsom, PA, USA) coupled with a CP-401 pH-meter (Elmetron, Zabrze, Poland), and each record was collected after 2 min of stabilization time.

### 3.11. High-Performance Liquid Chromatography

The LV concentrations in the degradation study were measured with the pharmacopoeial method [40]. The samples obtained in release experiment were analyzed with a Hitachi Primaide HPLC set (Hitachi HTA, Schaumburg, IL, USA) equipped with a Purospher^®^STAR RP-18 endcapped (5 μm) 250 × 4.6 mm column (Merck Millipore, Burlington, MA, USA). 0.025 M ammonium acetate solution with pH adjusted to 3.8 ± 0.2 with acetic acid mixed with methanol in a 95:5 ratio was employed as the mobile phase. Analysis was performed at 35 °C with 1 mL/min mobile phase flow. A Primaide 1410 UV detector (Hitachi HTA) was employed to detect analytes at 277 nm wavelength. Each sample was evaluated two times; reported concentrations are expressed as the mean of duplicates.

## 4. Conclusions

The influence of the reaction temperature and catalyst concentration on the reaction rate of LV-initiated homopolymerization of *ε*-CL and copolymerization of *ε*-CL and LA catalyzed by SO was investigated. Both the temperature and SO concentration increase resulted in increased reaction rates. The activation energies for the homopolymerization and copolymerization reactions were 95.86 ± 0.79 kJ mol^-1^ and 63.08 ± 14.27 kJ mol^−1^, respectively. The elevated reaction temperatures and high SO concentration promoted intramolecular transesterification side reactions resulting in poor reaction control and the broadening of molecular weight distribution. The variability of the crystallinity level, chemical composition, and molecular weight significantly influenced the drug release profile of LV from submicron particles composed of synthesized conjugates. The concentration of drug released from low molecular weight conjugates achieved plateau approximately after one month of exposition to physiological-like conditions. High molecular weight conjugates exhibited a distinct delay in drug release with a subsequent burst release. The molecular weight of conjugates is an important determining factor of the degradation mechanisms of the particles.

## Figures and Tables

**Table 1:** Summary of mass spectra matching with isotopic distributions simulated for particular polymeric structures. Acronyms include information about the type of lamivudine-initiated polymerization (LVCL: homopolymerization, LVCO: copolymerization), applied reaction temperature (100, 115, 130, 145 °C) and the employed catalyst to initiator ratio (1:3, 1:4, 1:7, 1:28). PLA: polylactic acid, PCL: poly(*ε*-caprolactone).

Synthesis	Structure Matched to Simulation *	Adduct/s
LVCO115_1:28	Conjugated poly(d,l-lactide-*co*-*ε*-caprolactone)chain	H^+^
	Conjugated PLA chain	H^+^
	Conjugated PCL chain	H^+^
	PLA chain	K^+^
LVCO115_1:7	Conjugated poly(d,l-lactide-*co*-*ε*-caprolactone)chain	H^+^
	Conjugated PLA chain	H^+^
	Conjugated PCL chain	H^+^
LVCO115_1:4	Conjugated poly(d,l-lactide-*co*-*ε*-caprolactone) chain	H^+^
	PLA chains	Na^+^
	Conjugated PLA chain	H^+^
LVCO115_1:3	Conjugated poly(d,l-lactide-*co*-*ε*-caprolactone) chain	H^+^
	Conjugated PLA chain	H^+^
LVCL115_1:28	Conjugated PCL chain	H^+^
LVCL115_1:7	Conjugated PCL chain	Na^+^, H^+^
LVCL115_1:4	Conjugated PCL chain	H^+^
LVCL115_1:3	Conjugated PCL chain	H^+^
LVCO100_1:7	Conjugated poly(d,l-lactide-*co*-*ε*-caprolactone) chain	H^+^
	Conjugated PLA chain	H^+^
LVCO130_1:7	Conjugated poly(d,l-lactide-*co*-*ε*-caprolactone) chain	H^+^
	Conjugated PCL chain	H^+^
LVCO145_1:7	Conjugated poly(d,l-lactide-*co*-*ε*-caprolactone) chain	H^+^
	Conjugated PLA chain	H^+^
LVCL 100_1:7	Conjugated PCL chain	H^+^
	PCL chain	Na^+^
LVCL130_1:7	Conjugated PCL chain	H^+^
	PCL chain	Na^+^
LVCL145_1:7	Conjugated PCL chain	H^+^
	PCL chain	Na^+^

* Simulated and observed isotopic distributions were matched with tolerance of 0.05 Da.

**Table 2:** Rate constants with standard errors for each performed LV-initiated ring-opening polymerization (ROP); a detailed explanation of the synthesis abbreviations is included in the Table 1 header.

Synthesis	Apparent Rate Constant k (h^−1^)	Standard Error	R-Square
LVCO115_1:28	0.159	0.007	0.98
LVCO115_1:7	0.440	0.013	0.99
LVCO115_1:4	0.750	0.036	0.98
LVCO115_1:3	0.759	0.050	0.97
LVCL115_1:28	0.090	0.007	0.96
LVCL115_1:7	0.958	0.057	0.98
LVCL115_1:4	1.229	0.155	0.94
LVCL115_1:3	2.566	0.190	0.98
LVCO100_1:7	0.125	0.004	0.99
LVCO130_1:7	0.962	0.069	0.96
LVCO145_1:7	1.069	0.156	0.87
LVCL 100_1:7	0.279	0.028	0.93
LVCL130_1:7	2.714	0.377	0.91
LVCL145_1:7	7.724	0.156	0.99

**Table 3:** Physical properties of conjugates obtained in all the conducted ROP procedures; a detailed explanation of the synthesis abbreviations is included in the Table 1 header.

Synthesis	M_n_ (kDa)	M_w_ (kDa)	PDI	Cryst. (%)	T_m_ (˚C)	LA:*ε*-CL Ratio	Conv. (%)
LVCL100_1:7	1.54	2.4	1.56	66	55	-	80%
LVCL115_1:7	4.59	7.08	1.54	60	60.8	-	100%
LVCL130_1:7	3.84	8.04	2.09	61	60.1	-	100%
LVCL145_1:7	5.47	11.76	2.15	59	62.8	-	100%
LVCL115_1:28	0.99	1.28	1.3	62	54.7	-	39%
LVCL115_1:4	3.87	6.78	1.9	56	61.5	-	100%
LVCL115_1:3	6.01	10.4	1.73	60	62.8	-	100%
LVCO100_1:7	0.88	1.23	1.4	0	-	1.3166	51%
LVCO115_1:7	2.16	4.23	1.96	3	-	0.891	88%
LVCO130_1:7	2.78	5.27	1.89	0	-	0.8328	99%
LVCO145_1:7	3.16	6.21	1.96	0	-	0.9156	99%
LVCO115_1:28	0.92	1.33	1.45	0	-	3.8382	52%
LVCO115_1:4	2.38	4.92	1.97	0	-	0.662	97%
LVCO115_1:3	3.12	6.15	2.07	7	-	0.7278	96%

**Table 4:** Characteristics of obtained submicron conjugate-based particles; a detailed explanation of the synthesis abbreviations is included in the Table 1 header.

Preparation	Conjugate	Hydrodynamic Diameter (nm)	PDI_Hd_
LVPLACL_DDS_1	LVCO100_1:7	389.6 ± 5.4	0.46 ± 0.02
LVPLACL_DDS_2	LVCO115_1:28	380.9 ± 18.6	0.51 ± 0.06
LVPLACL_DDS_3	LVCO145_1:7	440.8 ± 11.1	0.39 ± 0.02
LVCL_DDS_1	LVCL115_1:28	353.4 ± 4.6	0.45 ± 0.05
LVCL_DDS_2	LVCL115_1:4	506.9 ± 6.964	0.26 ± 0.02

**Table 5:** Parameters of each evaluated homopolymerization and copolymerization reaction; a detailed explanation of the synthesis abbreviations is included in the Table 1 header.

Synthesis	SO: Initiator: LA: *ε*-CL Molar Ratio	Reaction Temperature (°C)	Initiator	Monomer
ACCL	1:31:0:1948	165	AC	*ε*-CL
CLARCL	1:12:0:1948	115	CLAR	*ε*-CL
RIFCL	1:12:0:1948	115	RIF	*ε*-CL
LVCL100_1:7	1:7:0:438	100	LV	*ε*-CL
LVCL115_1:7	1:7:0:438	115	LV	*ε*-CL
LVCL130_1:7	1:7:0:438	130	LV	*ε*-CL
LVCL145_1:7	1:7:0:438	145	LV	*ε*-CL
LVCL115_1:28	1:28:0:1753	115	LV	*ε*-CL
LVCL115_1:4	1:4:0:250	115	LV	*ε*-CL
LVCL115_1:3	1:3:0:175	115	LV	*ε*-CL
LVCO100_1:7	1:7:77:204	100	LV	*ε*-CL, LA
LVCO115_1:7	1:7:77:204	115	LV	*ε*-CL, LA
LVCO130_1:7	1:7:77:204	130	LV	*ε*-CL, LA
LVCO145_1:7	1:7:77:204	145	LV	*ε*-CL, LA
LVCO115_1:28	1:28:310:818	115	LV	*ε*-CL, LA
LVCO115_1:4	1:4:44:116	115	LV	*ε*-CL, LA
LVCO115_1:3	1:3:31:81	115	LV	*ε*-CL, LA
